# Outcomes of Cryoballoon Ablation in High- and Low-Volume Atrial Fibrillation Ablation Centres: A Russian Pilot Survey

**DOI:** 10.1155/2015/591603

**Published:** 2015-11-12

**Authors:** Evgeny N. Mikhaylov, Dmitry S. Lebedev, Evgeny A. Pokushalov, Karapet V. Davtyan, Eduard A. Ivanitskii, Anatoly A. Nechepurenko, Alexey Ya. Kosonogov, Grigory V. Kolunin, Igor A. Morozov, Sergey A. Termosesov, Evgeny B. Maykov, Dmitry N. Khomutinin, Sergey A. Eremin, Igor M. Mayorov, Alexander B. Romanov, Vitaliy V. Shabanov, Victoria Shatakhtsyan, Viktor Tsivkovskii, Amiran Sh. Revishvili, Evgeny V. Shlyakhto

**Affiliations:** ^1^Arrhythmia Department, Federal Almazov North-West Medical Research Centre, Saint Petersburg 197341, Russia; ^2^Neuromodulation Unit, Federal Almazov North-West Medical Research Centre, Saint Petersburg 197341, Russia; ^3^Center of Interventional Cardiology, State Research Institute of Circulation Pathology, Novosibirsk 630055, Russia; ^4^National Research Center for Preventive Medicine, Moscow 101990, Russia; ^5^Federal Center for Cardiovascular Surgery, Krasnoyarsk 660020, Russia; ^6^Federal Center for Cardiovascular Surgery, Astrakhan 414011, Russia; ^7^Electrophysiology Department, City Hospital #5, Nizhny Novgorod 603005, Russia; ^8^Electrophysiology Department, Tyumen Cardiology Center, Tyumen 625026, Russia; ^9^Electrophysiology Department, Research Institute of Cardiology, Saratov 410028, Russia; ^10^Cardiovascular Surgery Department, City Hospital #12, Moscow 115516, Russia; ^11^Department of Clinical Electrophysiology, Russian Cardiology Research and Production Center, Moscow 121552, Russia; ^12^Tyumen Regional Hospital #1, Tyumen 625032, Russia; ^13^Republican Clinical Hospital of the Republic of Tatarstan, Kazan 420064, Russia; ^14^Botkin City Hospital, Moscow 125284, Russia; ^15^Bakulev Scientific Center of Cardiovascular Surgery, Moscow 121552, Russia; ^16^Institute of Heart and Vessels, Federal Almazov North-West Medical Research Centre, Saint Petersburg 197341, Russia

## Abstract

*Purpose*. The results of cryoballoon ablation (CBA) procedure have been mainly derived from studies conducted in experienced atrial fibrillation (AF) ablation centres. Here, we report on CBA efficacy and complications resulting from real practice of this procedure at both high- and low-volume centres.* Methods*. Among 62 Russian centres performing AF ablation, 15 (24%) used CBA technology for pulmonary vein isolation. The centres were asked to provide a detailed description of all CBA procedures performed and complications, if encountered.* Results*. Thirteen sites completed interviews on all CBAs in their centres (>95% of CBAs in Russia). Six sites were high-volume AF ablation (>100 AF cases/year) centres, and 7 were low-volume AF ablation. There was no statistical difference in arrhythmia-free rates between high- and low-volume centres (64.6 versus 60.8% at 6 months). Major complications developed in 1.5% of patients and were equally distributed between high- and low-volume centres. Minor procedure-related events were encountered in 8% of patients and were more prevalent in high-volume centres. Total event and vascular access site event rates were higher in women than in men.* Conclusions*. CBA has an acceptable efficacy profile in real practice. In less experienced AF ablation centres, the major complication rate is equal to that in high-volume centres.

## 1. Introduction

Cryoballoon ablation (CBA) for pulmonary vein (PV) isolation has become a treatment used worldwide for patients with atrial fibrillation (AF). As with other “single-shot device” technologies, CBA aims to simplify and shorten the PV ablation procedure in patients with recurrent arrhythmia [1]. The number of electrophysiology centres is growing continuously, and some sites may not be well experienced in complex procedures when they launch AF ablation programmes [2]. It is not yet well understood which PV isolation technology is best advised for centres just starting to offer interventional AF management, since different energy sources and types of ablation have different learning curves and complication rates [3].

We sought to investigate whether CBA started in centres not previously experienced in AF ablation procedures might be safe and effective. Therefore, the aim of this study was to describe the techniques and results of CBA PV isolation in the Russian Federation and to compare AF ablation results between high- and low-volume AF ablation centres beginning to use this cryoablation technology.

## 2. Methods

According to the information obtained from a national electrophysiology centres registry, among 62 centres performing AF ablation between 2010 and 2014 in the Russian Federation, 23 sites used catheter cryotechnology. Of those centres, 15 (24%) performed cryoballoon PV isolation. These centres were contacted and asked to participate in a national survey. Fourteen centres responded, and a general questionnaire was provided to centres' representatives. Thirteen (87%) centres returned a fully completed questionnaire. In a second step, an additional questionnaire was sent to centres that had reported procedure-related adverse events.

The study was approved by the Federal North-West Medical Research Centre ethics committee. The study was conducted under the auspices of the Russian Society of Cardiology and the Russian Scientific Society of Clinical Electrophysiology, Arrhythmology and Cardiac Pacing.

### 2.1. General Questionnaire

The general questionnaire consisted of 8 sections and 74 questions in total (Supplementary Table 1 in Supplementary Material available online at http://dx.doi.org/10.1155/2015/591603): centre experience in catheter ablation (7 questions); centre experience in cryoballoon ablation (8 questions); cryoballoon ablation technique (28 questions); patient follow-up methods (8 questions); periprocedural anticoagulation (6 questions); CBA efficacy (3 questions); procedure-related events and complications (9 questions); recurrence specifications (5 questions).

In order to investigate a mean number of AF procedures per year and to classify the volume of AF ablation centres, responders were required to report measures collected during the 2 most recent years. Investigation of clinical outcomes (arrhythmia-free rate) was based on a 6-month follow-up period in the centres with an outpatient control programme. Additionally, the 12-month arrhythmia-free rate was analyzed separately, when available.

### 2.2. Additional Questionnaire upon Procedure-Related Events

Ten centres reporting procedure-related events were asked to provide additional detailed information regarding patient characteristics and further management (Supplementary Table 2). Additional questions were addressed to centres to clarify ambiguous responses and in order to obtain left atrial sizes, PV isolation verification techniques and successes, and so forth. All centres provided full responses to additional questions.

Major complications were classified as procedure-related events, requiring additional interventions and/or prolonging hospitalization. Other conditions were classified as procedure-related minor events: vascular access site problems (minor groin haematoma, etc.), transient phrenic nerve paresis/dysfunction with restoration during the index procedure, pericardial effusion without consequence, and so forth.

### 2.3. Statistical Analysis

Continuous variables were expressed as mean ± standard deviation and were compared using the *t*-test, if their distribution did not deviate significantly from the normal distribution (tested with the Kolmogorov-Smirnov test). If significant deviation from the normal distribution was found, continuous variables were expressed as the median within the interquartile range (IQR) and were compared using nonparametric tests (Mann-Whitney *U* tests and Fisher's exact test). Categorical variables were expressed as percentages and numbers. Weighted average percentages were provided for percentages taken from different totals. Percentages were compared using the “difference between proportions” function. A 2-tailed *p* < 0.05 indicated statistical significance. Analysis was performed using Statistica 6.0 software (StatSoft Inc., Tulsa, OK, USA).

## 3. Results

### 3.1. Centres' Characteristics

Average experience in catheter ablation among the 13 participating centres was 9.8 ± 5.7 years, with AF ablation programmes started on average 6.2 ± 3.4 years ago. The median number of ablation procedures was 2149 (IQR: 1330–4010) per centre.

More than 100 AF procedures per year were performed in 6 centres (median 202.6; IQR 142.3–493.4 AF cases per year), and they were classified as higher-volume AF ablation sites according to the 2012 HRS/EHRA/ECAS Expert Consensus Statement, which declared that better AF ablation outcomes are seen in centres with >100 cases [3]. The other 7 centres performed <100 (median 25.7; IQR: 13–89.4) AF cases per year and were classified as lower-volume AF ablation sites (Table 1). Additionally, safety and efficacy were calculated separately for the 3 most experienced centres, performing >400 AF ablations per year.

Furthermore, in order to investigate CBA outcomes in centres with different experience in all arrhythmia types ablation (not only AF), the sites were divided into low-ablation experience (1st quartile, ≤1330 ablations; 4 centres), medium-ablation experience (>1330 and ≤4010 procedures; 5 centres), and high-ablation experience (3rd quartile, >4010 procedures; 4 centres).

The median proportion of CBA procedures among all AF ablations was 12.5% (IQR: 4.5–26.9%) per centre.

The number of radiofrequency (RF) AF ablation procedures increased in 2013/2014 by 15% per month, while the absolute number of CBA procedures increased by 214%.

Two nonresponding centres were low-volume AF ablation sites. One centre performed 6 CBA procedures (data from the centre) and the other less than 20 (data from the company). Therefore, the survey data included 457 procedures, representing >95% of all CBA cases performed in the country.

### 3.2. Patient Characteristics

A single CBA procedure was performed in 457 patients (56.3% males; mean age 54.8 ± 5.4 years). In 428 (94%) patients, AF was paroxysmal, and in 29 (6%) it was persistent. In all patients, arrhythmia was refractory to at least one antiarrhythmic drug.

### 3.3. Ablation Procedure Characteristics

Ultrasound guidance for transseptal access was routinely used in 10 centres but used only in certain cases in 1 centre (5 transoesophageal, 4 intracardiac, and 2 either transoesophageal or intracardiac echo). Only 2 centres adopted intracardiac ultrasound guidance for controlling balloon positioning within PV ostia.

Double transseptal punctures were used in 7 centres and single punctures in 6. An additional circular diagnostic catheter was used in 8 centres. The number of transseptal punctures was strongly dependent on the use of the integrated Achieve (Medtronic, Minneapolis, MN, USA) circular catheter. Sites that adopted this catheter switched to one transseptal access.

All centres used the Arctic Front balloon (Medtronic, Minneapolis, MN, USA), since the second-generation balloon was not available in the Russian Federation until 2015. The balloon catheter was introduced into the left atrium via a 14-F transseptal steerable sheath (FlexCath, Medtronic, Minneapolis, MN, USA). Among the 13 centres, 12 used only 28 mm balloons, while 1 low-volume centre adopted exclusively 23 mm balloons (aiming to obtain a higher occlusion rate of all PVs). All centres reported that at least two freeze cycles of 300 s in duration were delivered to each PV.

In all participating centres, the endpoint of the CBA procedure was complete electrical disconnection of all PVs. RF touch-up applications, in order to complete PV isolation, were reported by 4 centres (in 5.1% (0–23%) of patients); operators preferred RF applications due to faster performance and lower cost of this technology in comparison with cryo touch-ups. No linear ablation in the left atrium was performed. Concomitant right atrial isthmus RF ablation was performed in patients with documented typical flutter. Bidirectional block in the PVs was the endpoint of the CBA in 9 centres, whereas in 4 centres operators aimed to achieve entrance block as the endpoint. According to the prespecified endpoint, isolation of all PVs was obtained in 89.3% of patients. A nonisolated PV was almost exclusively the right inferior PV.

The mean procedure time was 155.7 ± 35.7 min and the mean fluoroscopy time was 27.7 ± 10.2 min among all centres. Four high-volume centres provided fluoroscopy and total procedure times for the first 20 and all subsequent procedures: 36.0 ± 14.5 versus 31.0 ± 15.8 min and 191.3 ± 29.5 versus 158.5 ± 18.9 min, respectively (*p* > 0.05).

Implementation of the Achieve circular catheter was associated with a statistically significant decrease in the mean fluoroscopy and total procedure times: 29.8 ± 10.8 versus 26.3 ± 10.4 min and 165.4 ± 22.0 versus 141.2 ± 37.6 min, correspondingly, *p* < 0.05.

Periprocedural anticoagulation strategies were mixed in 12 centres; operators used bridge anticoagulation with low molecular weight heparin in 10 centres and uninterrupted Warfarin in 6. Periprocedural anticoagulation with novel oral anticoagulants (NOACs) was reported by 11 centres: Dabigatran was skipped 12–48 hours before the procedure and Rivaroxaban 12–24 hours before the procedure. One centre used an uninterrupted Warfarin strategy only.

### 3.4. Patient Follow-Up

Median hospital stay was 3 (1–6) days (IQR: 3-4). Regular patient checks were performed in 11 centres. Recurrence documentation was carried out by resting ECG registration and 24-hour Holter monitoring (every 1–6 months) in 11 sites; subcutaneous loop recorders were implanted in 2 centres (in 60% and 13% of patients in these centres). Regular personal visits to outpatient units were performed in 7 centres, and 9 centres additionally contacted patients by phone (every 3, 6, or 12 months).

Ten centres reported 6-month arrhythmia-free rates of 63.9% after paroxysmal AF CBA off antiarrhythmics, after a 3-month blanking period. Persistent AF CBA was performed in 2 centres with arrhythmia-free rates of 50% and 66.7% at 6 months off antiarrhythmics.

Six centres provided 1-year follow-up data with a mean arrhythmia-free rate of 64.1% (50–75.8%) off antiarrhythmic drugs. One of the two centres, which used implantable loop recorders for recurrence documentation, reported a 66% arrhythmia-free rate at one year (off antiarrhythmics).

Regular supraventricular tachycardia as a recurrence was encountered in 18 (4%) patients. In 11 (2.4%) patients, it was finally diagnosed as typical atrial flutter. Left atrial tachycardia (AT) was documented in 6 (1.3%) patients, and in 1 (0.2%) patient it was a macroreentrant AT.

Additionally, sites were asked to report the number of redo cases following CBA. Twelve centres provided data within 12 months of follow-up: a redo procedure was performed in 25 patients (7.1% of patients out of 12 centres); RF PV reisolation was carried out in all cases. The centres reported that in redo cases they used the point-by-point RF technique instead of CBA due to the lower cost of the procedure and possible mapping necessity for non-PV tachycardias.

### 3.5. Procedure-Related Events

Major complications developed in seven (1.5%) patients. Cardiac tamponade was encountered in 4 patients, all of which were managed by percutaneous drainage without further sequelae. Ischaemic stroke occurred in 2 patients: 1 patient completely recovered, while the other patient had partial permanent disability. Persistent phrenic nerve palsy (>6 months) was reported in 1 patient. No atrioesophageal fistulas or deaths within 60 days were reported.

Minor procedure-related events were encountered in 37 (8%) patients and included transient phrenic nerve paresis/dysfunction, haemoptysis, and pericardial effusion (Table 2). In the centre which adopted the 23 mm balloon, one transient phrenic nerve paresis was seen. In 5 patients with haemoptysis, symptoms resolved spontaneously within 2 days. Additional questions were addressed to operators regarding this complication. It was noted that the minimum temperature during ablation in these patients was from −53 to −67°C. No specific reason for this adverse event was identified; a 28 mm balloon was used in these patients.

The total procedure-related event rate was higher in women than in men (12% versus 4.9%; *p* < 0.05), mainly due to the higher prevalence of vascular access site minor events in females (6% in women versus 0% in men; *p* < 0.05).

### 3.6. Cryoballoon Ablation in High- versus Low-Volume AF Ablation Centres

In compliance with the criteria of centre volume, two groups had significant differences in the number of total catheter-ablation procedures and number of AF procedures (Table 2). On the other hand, there was only a borderline difference between the durations of high- and low- volume centres' AF ablation programmes (*p* = 0.05). The mean proportion of CBAs among all AF ablation cases was higher in low-volume than in the high-volume centres. A higher proportion of male patients were present in the high-volume centres. There was no statistical difference in the routine use of ultrasound guidance during the procedure or in mean fluoroscopy and total procedure times. Isolation of all PVs was more frequently achieved in high-volume centres (Table 2), and the right inferior PV was the only nonisolated vein in some patients in low-volume centres.

Mid-term (6 months) freedom from any arrhythmia was comparable between high- and low-volume AF ablation centres.

Major complication rates were similar in high- and low-volume centres (1.4% versus 2.0%, *p* > 0.05). Minor procedure-related events were more frequently seen in high-volume centres than in low-volume AF ablation centres (9.9% versus 1.0%, *p* = 0.001). Due to the prevalence of minor events, the total number of all adverse events was higher in the high-volume centres. However, there were no differences in specific conditions between the groups (Table 2).

When calculated in the 3 highest-volume centres (>400 AF cases per year; 205 CBA procedures in total), CBA success and major complication rates did not differ from those in the low-volume centres (68.9% and 2.4%, resp., *p* > 0.05). However, minor adverse events were reported more frequently (8.3%; *p* = 0.04).

### 3.7. Cryoballoon Ablation Outcomes in Centres with Different Experience in All Arrhythmia Types Ablation

When centres were classified according to experience in ablation of all arrhythmia types, it was found that minor procedure-related events were still more frequently seen in high-experienced centres, but in comparison only with medium-experienced centres (Table 3). There were no differences in arrhythmia-free rates and major complication rates between high- and low-experienced centres.

## 4. Discussion

In low-volume AF ablation centres, where operators have experience in simple ablation procedures, CBA is a safe procedure with success rates comparable to those obtained in higher-volume AF centres. Major complications of CBA are infrequently encountered in real practice and rarely lead to permanent disability. Our results underline that CBA has a fast and reproducible learning curve in both high- and low-volume AF ablation centres.

### 4.1. Cryoballoon Ablation Procedure Characteristics

As shown in this study, the number of CBA procedures is growing, especially in centres that have recently started AF ablation programmes. A similar increase in the number of CBAs has been reported in a German AF ablation registry [4].

In accordance with the results of previous studies, CBA has a short learning curve and usually about 20–50 procedures are required to reach a plateau [5, 6]. In this study, no significant decline in fluoroscopy and procedure times was observed after the first 20 CBA procedures, most likely due to the limited data provided. However, implementation of an integrated diagnostic circular catheter was associated with a significant reduction in fluoroscopy and procedure durations and these findings echo previous reports [7].

Fluoroscopy and total procedure times were shorter in our study than those in previous reports [1, 8, 9]. It is suggested that previous personal experience in simpler ablation procedures might play an important role in this result.

Another interesting finding was the low rate of additional touch-up applications required for PV isolation, especially in the low-volume centres. Although the intended endpoint of CBA was complete PV isolation in all centres, the right inferior PV was sometimes left nonisolated by operators with less experience in RF left atrial ablation, instead of performing aggressive attempts with additional equipment to complete isolation. Nevertheless, when comparing recurrence rates in the high- and low-volume sites, this does not seem influencing the mid-term results significantly, suggesting that the right inferior PV might play a minor role in arrhythmogenesis.

Periprocedural anticoagulation strategy was mixed in the majority of centres and depended on what kind of anticoagulant was used in patients prior to ablation. We did not receive information on specific anticoagulation strategies in patients with cardiac tamponade or vascular adverse events. Previous studies have suggested that there is no significant difference between the use of uninterrupted Warfarin and the use of NOACs [10]. Several randomized studies addressing this issue are underway.

### 4.2. Cryoballoon Ablation Efficacy

The mid-term arrhythmia-free rate (at 6 and 12 months of follow-up) in our survey was slightly lower when compared with previous AF ablation surveys and studies [1, 4, 8, 11–13]. We suggest that this could be related to the fact that all participating centres were relatively inexperienced in the use of the cryotechnology. Moreover, only the first-generation balloon was used in all centres.

In our study, two centres reported success rates of persistent AF CBA. This form of AF was present only in 6% of patients treated; therefore, we are not able to show any reliable comparison with the group of paroxysmal AF. The results of CBA in this group were promising; however, we should appreciate the small size of this patient subgroup.

Regular supraventricular tachycardia as a recurrence has been found in 4% of patients and was a macroreentrant left AT in only 1 patient. The low incidence of AT and reentrant arrhythmia after CBA was in accordance with previously published reports [8, 14].

### 4.3. Ablation Results Using First- and Second-Generation Cryoballoons

Importantly, our survey results reflect experience with the first-generation cryoballoon. Currently, a second-generation cryoballoon is available in many countries and several studies have compared the efficacy of the first- and second-generation devices. These reports show that the arrhythmia-free rate after ablation using the newer balloon yields significantly better results ranging around 80–84% [15–17].

It has been also shown that a single application approach using the newer balloon reduces cryoablation and fluoroscopy times while achieving excellent results [18, 19]. All these findings indicate that the newer device is more powerful when compared to the first-generation balloon.

The main idea of this study was to compare AF CBA in different volume centres, and the cryoablation procedure performed with either the first- or second-generation balloons is still a “one-shot” device technology for achieving PV isolation. Moreover, we believe that the performance of the ablation procedure was more reproducible among the centres, since all of them used the same first-generation balloon.

### 4.4. Procedure-Related Events

It should be acknowledged that, in the low-volume AF ablation centres, personal operators' experience in simple ablation procedures was considerable and some operators had performed >1000 ablation procedures for supraventricular and ventricular tachyarrhythmias before launching AF ablation programmes. Therefore, the results of this study should be interpreted in light of previous experience of operators regarding vascular access and intracardiac manipulations.

Cardiac tamponade occurred in 0.87% of cases. Considering the fact that about a quarter of CBAs were performed in low-volume AF ablation centres, this number appears low [20]. Since 61% of centres used ultrasound guidance for transseptal access, it is possible that this significantly decreased the risk of cardiac perforation. Moreover, one would expect that the risk of cardiac perforation during CBA might be lower than during RF ablation, since cryolesion cannot induce tissue overheating and wall disruption. In a study of about 35,000 AF ablation procedures, it was found that a considerable number of left atrial perforations occurred during RF energy delivery in the left atrium [20].

In patients with haemoptysis, no reasons for its development were identified and questions regarding the aetiology of this condition remained unanswered. Several reports of haemoptysis after CBA procedures have been published before and the authors suggested several potential causes, including lung tissue injury by distal balloon inflation, direct injury by a guidewire, or parenchymal pulmonary infarction due to acute PV occlusion [21]. We noted that the minimum ablation temperature achieved in these patients was low (−53 to −67°C), and this is in accordance with previous reports [22].

Our study showed low incidence of persistent (>6 months) phrenic nerve palsy. We believe that this was related to the almost exclusive use of a first-generation 28 mm balloon and close supervision of phrenic nerve function during right PV ablation by all operators. Previous studies have identified an association between a higher proportion of phrenic nerve damage with a smaller balloon size and the second-generation device [5, 15].

Gastroparesis has been earlier suggested as one of the underreported AF ablation complications [23]. It should be acknowledged that no cases of gastroparesis were documented in our survey.

A higher proportion of procedure-related minor events were found in higher-volume AF ablation centres. This finding is further confirmed by evaluation of CBA outcomes in centres with different experience in all arrhythmia types ablation. This can be partly explained by the fact that, in the lower-volume AF ablation centres, venous access was performed by well experienced vascular puncture operators. The higher-volume centres were mainly academic teaching centres, where preparations for ablation procedures were carried out by younger fellow physicians.

Our findings show that female gender patients might be associated with higher incidence of minor procedure-related events, mainly vascular access site complications. It is difficult to explain such prevalence in females, since we had no access to individual clinical data of patients without adverse events. Theoretically, a higher proportion of vascular access site problems in women might be associated with more increased body mass index or more variable femoral vein course. No gender difference was found in major complications; however, this could be explained by a limited number of cases for such analysis.

### 4.5. Clinical Implications

It seems that very experienced operators mainly prefer RF catheter ablation with an acceptable efficacy rate and low incidence of major complications. However, RF PV isolation requires substantial operator experience, with previous work having been closely supervised, and the failure rates and number of complications during the initial stages of gaining experience are high [2, 3]. In this regard, CBA may be advocated for low-volume AF ablation centres, or centres starting their AF ablation programme, as a safe and equally effective procedure in properly selected patients.

## 5. Study Limitations

There are several limitations that may influence the results of this study. The study data are mainly derived from questionnaires, and the study is a survey in nature. A limited number of centres should be acknowledged. However, this was a national survey and the participating sites represented >86% of all sites performing CBA in the country. Another limitation is that there was a difference in arrhythmia recurrence detection methods (Holter monitoring, implantable loop recorder interrogation) and a difference in time intervals between follow-up visits in the centres.

We had no access to detailed clinical information regarding patients without complications; therefore, we were not able to analyze predictors associated with a favourable outcome or complications.

Since there were more trainees in the high-volume centres, this might have also affected long term results in these centres. However, it is known that trainees were closely supervised by very experienced operators during the main stage of the procedure.

## 6. Conclusion

In conclusion, CBA can be safely performed in low-volume AF ablation centres without compromising efficacy.

## Supplementary Material

Supplementary Table 1 represents the general questionnaire upon centre experience in catheter ablation, cryoballoon ablation technique, patient characteristics, procedure data, and follow-up (translation from Russian).
Supplementary Table 2 represents additional questionnaire regarding procedure-related events (translation from Russian).

## Figures and Tables

**Table 1:** AF ablation centres' characteristics.

Centre code	Total number of catheter ablations	Total number of CBAs	Total AF ablations	AF ablations/year^*∗*^	High AF volume center
1	12001	109	5232	1011	+
2	1596	71	568	202	+
3	4298	69	1532	411	+
4	3416	55	353	123	+
5	2149	33	284	89	−
6	7434	27	2364	575	+
7	4010	24	1237	161	+
8	2445	18	52	15	−
9	235	13	40	25	−
10	1330	13	16	6	−
11	912	10	217	60	−
12	1750	8	43	3	−
13	128	7	26	12	−

Total	41704	457	11964	2693	46%

^*∗*^Calculated for the 2 last years; AF: atrial fibrillation; CBA: cryoballoon ablation.

**Table 2:** Characteristics of procedure results in high- and low-volume AF ablation centres.

Parameter	Overall	High-volume AF ablation centres, *N* = 6	Low-volume AF ablation centres, *N* = 7	*p*
Mean experience in catheter ablation, years	9.8 ± 5.7	9.7 ± 4.2	10.9 ± 6.3	0.7
Median number of catheter-ablation procedures	2149 (IQR: 1330–4010)	4010 (IQR: 2782.5–5866)	1750 (IQR: 912–2445)	0.008
Median number of catheter-ablation procedures per year	266.7 (IQR: 97.1–569.3)	569.3 (IQR: 266.7–842.8)	114 (IQR: 78.3–267.3)	0.005
Median experience in AF ablation, years	5 (IQR: 4–8)	6 (IQR: 5–10.5)	5 (IQR: 4–8)	0.05
Median number of AF ablation procedures	284 (IQR: 43–1237)	1237 (IQR: 460.5–1948.5)	52 (IQR: 40–284)	0.001
Median number of AF ablation procedures per year	89.4 (IQR: 15–202.6)	202.6 (IQR: 142.3–493.4)	25.7 (IQR: 13–89.4)	0.001
Mean number of CBAs	35.2 ± 29.0	59.1 ± 31.7	14.6 ± 8.9	0.004
Proportion of CBA among all AF cases	12.5% (IQR: 4.5–26.9)	4.5% (IQR: 2–12)	18.6% (IQR: 4.6–13)	0.01
Mean age, years	54.8 ± 5.4	53.6 ± 2.3	55.9 ± 7.1	0.46
Gender, % of males	56.3%	62.8%	50.8%	0.002
Left atrial diameter, mm	41.9	41.8	42.1	0.33
Routine echo guidance, number of centers	10	4	6	1.05
Periprocedural anticoagulation strategy, centers				
Uninterrupted Warfarin	6	2	4	0.59
Bridge anticoagulation	10	5	5	1.0
NOACs	11	5	6	1.0
Fluoroscopy time, min	27.7 ± 10.2	24.6 ± 9.9	33.8 ± 9.5	0.34
Procedure time, min	155.7 ± 35.7	154.5 ± 28.4	156.8 ± 44.8	0.91
RF touch-up applications, centers	4	3	1	0.19
Isolation of all PVs, % of patients	89.3%	97%	79.4%	<0.001
Arrhythmia-free rate at 6 months	63.9%	64.6%	60.8%	0.96
Major complications	7 (1.5%)	5 (1.4%)	2 (2.0%)	0.66
Tamponade	4 (0.87%)	3 (0.84%)	1 (0.98%)	1.0
Stroke	2 (0.4%)	2 (0.56%)	0	1.0
Persistent phrenic nerve palsy	1 (0.2%)	0	1 (0.98%)	1.0
Minor procedure-related events	37 (8%)	35 (9.9%)	2 (2.0%)	0.007
Pericardial effusion	1 (0.2%)	1 (0.28%)	0	1.0
Transient phrenic nerve paresis	23 (5%)	21 (5.9%)	2	0.08
Vascular complications	8 (1.8%)	8 (2.2%)	0	0.21
Arteriovenous fistula	3 (0.65%)	3 (0.84%)	0	1.0
Femoral artery pseudoaneurysm	5 (1%)	5 (1.4%)	0	0.59
Transient hemoptysis	5 (1%)	5 (1.4%)	0	0.59
Total procedure-related events	44 (9.6%)	40 (11.3%)	4 (3.9%)	0.03

AF: atrial fibrillation; CBA: cryoballoon ablation; IQR: interquartile range; NOACs: novel oral anticoagulants; RF: radiofrequency.

**Table 3:** CBA procedure outcomes in centres with low-, medium-, and high-catheter-ablation experience (all types of arrhythmia ablations).

Parameter	I Low ablation experience (4 centres)	II Medium ablation experience (5 centres)	III High ablation experience (4 centres)	*p*		
				Between I and II	Between II and III	Between I and III
Median number of catheter-ablation procedures	573 (IQR: 208–1016)	2445 (IQR: 2149–3416)	5866 (IQR: 3622–8575)	0.69	0.01	0.007
Median number of AF ablation procedures	33 (IQR: 23–84)	284 (IQR: 52–353)	1948 (IQR: 1291–3079)	0.02	0.02	<0.001
Mean number of CBAs	11 (IQR: 9–13)	24 (IRQ: 18–33)	69 ± 33.5	0.01	0.25	0.002
Total number of CBAs	43	138	276	NA	NA	NA
Fluoroscopy time, min	30.8 ± 11.7	29.9 ± 3.0	25.0 ± 12.3	0.08	0.05	0.97
Procedure time, min	165.0 ± 41.0	159.2 ± 43.4	142.8 ± 27.6	0.92	0.48	0.53
Arrhythmia-free rate at 6 months, %	66.6%	56.2%	67.5%	0.11	0.05	0.87
Major complications, *N* (%)	1 (2.3%)	1 (0.7%)	5 (1.8%)	0.42	0.67	0.58
Minor procedure-related events, *N* (%)	2 (4.6%)	4 (2.9%)	26 (9.4%)	0.63	0.015	0.49
Total procedure-related events, *N* (%)	3 (6.9%)	5 (3.6%)	31 (11.2%)	0.40	0.009	0.60

AF: atrial fibrillation; CBA: cryoballoon ablation; IQR: interquartile range.
