# Supplementary material for: Outcomes of Cryoballoon Ablation in High- and Low-Volume Atrial Fibrillation Ablation Centres: A Russian Pilot Survey
Source: Biomed Res Int. 2015 Nov 12;2015:591603. doi: 10.1155/2015/591603 (PMC4659951; doi:10.1155/2015/591603)
Supplement: Supplementary file 1 — Supplementary Table 1 represents the general questionnaire upon centre experience in catheter ablation, cryoballoon ablation technique, patient characteristics, procedure data, and follow-up (translation from Russian). Supplementary Table 2 represents additional questionnaire regarding procedure-related events (translation from Russian). [file 591603.f1.pdf]

SUPPLEMENTARY TABLE 1

General cryoballoon ablation questionnaire (translated from Russian)

**GENERAL QUESTIONS REGARDING CENTRE EXPERIENCE IN CATHETER ABLATION**									
[1]When your centre started tachyarrhythmia ablation?		[2]When your centre started AF ablation?		[3]Total number of all arrhythmia catheter ablations performed in your centre (before 10.2014)		[4]Total number of AF (RF+cryo) performed in your centre (before 10.2014)?			
[5]Total number of AF ablations (RF+cryo) performed in your centre in 2013		[6] Total number of AF ablations (RF+cryo) performed in your centre in 2014 (before 10.2014)		[7]When your centre started CBA?					
**QUESTIONS REGARDING CRYOBALLOON ABLATION EXPERIENCE**									
[8]Number of CBAs performed in your centre		[9]Of them for paroxysmal AF		[10] Of them for persistent AF		[11]N of males		[12]Mean age	
[13]Number of CBAs performed in 2013		[14]Number of CBAs performed in 2013 (before 10.2014)?		[15]Number of operators performing CBA					
**CRYOBALLOON ABLATION PARAMETERS**									
[16]Do you use Arctic Front balloons (yes\no)?		[17]Do you use 23 mm balloons (yes\no + %)?		[18] Do you use 28 mm balloons (yes\no +%)?		[19]Do you use the Achieve catheter? (yes\no)		[20]Do you use the Advance balloon (yes\no +%)	
[21]Number of transseptal punctures (with/without Achieve)		[22]Do you use other circular diagnostic catheter (with/without Achieve)?		[23]Do you use ultrasound guidance (routinely/only sometimes)?		[24]TEE (yes\no)?		[25]ICE (yes\no)	

[26]Ultrasound only for transseptal access (NA\yes\no)		[27] Ultrasound for balloon positioning as well (+doppler) (NA\yes\no)		[28]Mean fluoro time		[29]Mean fluoro time with Achieve/without Achieve (min)		[30] Mean fluoro time with Advance (min)	
[31] Mean fluoro time for first 20 procedures		[32]First 20-50 procedures		[33]Mean procedure time (min)		[34]Procedure time without Achieve (min)		[35]Procedure time with Achieve (min)	
[36]Procedure time with Advance (min)		[37]Mean procedure time for first 20 procedures		[38]First 20-50 procedures					
[39]Additional touch-up applications for PV isolation (Arctic Front) (%)		[40]Additional touch-up applications for PV isolation (Advance) (%)		[41]Touch-ups using RF (yes\no)		[42] Touch-ups using cryo (yes\no)		[43]RF ablation for additional linear lesions during the same procedure (except typical flutter) (%)	0

FOLLOW-UP (please consider a 3-month blanking period)									
[44]Duration of hospital stay (days)		[45]Do you routinely perform patient follow-up after CBA? (yes\no)		[46]24-hour Holter how frequently?		[47]48-72-hour Holter how frequently?		[48]Implantable loop recorder? (% of patients after CBA)	
[49]Rest ECG how frequently?		[50]Phone contact how frequently?		[51]Patients visit an outpatient unit? How frequently?					
**PERIPROCEDURAL ANTICOAGULATION (CBA only)**									
[52]Bridge anticoagulation routinely (yes/no)?		[53]Uninterrupted Warfarin (yes\no)?		[54]Non-vitamin K oral anticoagulants (NOACs)?		[55]Dabigatran – hours of skip (if used)		[56]Rivaroxaban - hours of skip (if used)	
[57]Apixaban – hours of skip (if used)									
**ARRHYTHMIA-FREE RATE AFTER CBA at 6/12 months – OFF AADs (no recurrence registered on ECG)**									
[58]Paroxysmal AF? (6/12 months, %)		[59]Persistent AF? (6/12 months, %)		[60]Long-standing persistent AF? (6/12 months, %)					

CBA PROCEDURE-RELATED ADVERSE EVENTS									
[61]Tamponade (number)		[62]Big hematoma (number)		[63]Arterio-venous fistula (number)		[64]Pulsating hematoma (number)		[65]Stroke (number)	
[66]Atrioesophageal fistula (number)		[67]Phrenic nerve paresis (any type and duration) (number)		[68]Death within 60 days after CBA (number)		[69]Pericardial effusion (number)		ANY OTHER	
**ATRIAL TACHYCARDIA AS A RECIURRENCE AFTER CBA**									
[70]Regular atrial tachycardia as a recurrence (number of patients)		[71]Of them typical atrial flutter (number)		[72]Number of patients with required redo ablation due to regular atrial tachycardia		[73]Of those pts with tachycardia – how many with left atrial?		[74]Number of patients with left atrial macro reentrant tachycardia during a redo procedure	

SUPPLEMENTARY TABLE 2

Additional questionnaire regarding procedure-related adverse events (translated form Russian)

**Tamponade**								
							**Gender**	**Age, years**
Patient-1	Possible reason and management							
Patient-2	Possible reason and management							
Please add more rows if required								
**Vascular adverse events**								
**Arteriovenous fistula**	Patient-1 - management							
	Patient-2 – management							
	Please add more rows if required							
**Pulsating hematoma**	Patient-1 – management							
	Patient-2 - management							
	Please add more rows if required							
**Phrenic nerve paresis**								
Patient-1	Transient? When recovered?							
Patient-2	Transient? When recovered?							
Please add more rows if required								
**Stroke**								
Patient-1	Ischemic/Hemorragic		When occurred?		Information about management and recovery			
Patient-2	Ischemic/Hemorragic		When occurred?		Information about managemen			

					t and recovery			
Please add more rows if required								
**Hemoptysis**								
Patient-1	Severe/mod arate/mild		When occurred?		Information about managemen t and recovery			
Please add more rows if required								
Please add any other information regarding procedure-related adverse events not indicated above								
